# Simulating Water Quality Benefits of Conservation Practices Using ArcAPEX in a Northeastern Arkansas Agricultural Watershed

**DOI:** 10.1007/s00267-026-02565-3

**Published:** 2026-08-05

**Authors:** Arjun Thapa, Niroj Aryal, Michele L. Reba, Anna Pieri, Geoffrey K. Payne

**Affiliations:** 1 North Carolina Agricultural and Technical State University, Greensboro, NC USA; 2 USDA-ARS, Delta Water Management Research Unit, Jonesboro, AR USA; 3 Division of Agriculture, University of Arkansas, Jonesboro, AR USA

**Keywords:** APEX model, Cover crops, Filter strips, Conservation practices, Watershed modeling, Uncertainty and reliability analysis

## Abstract

Predicting water quantity and quality from agricultural watersheds is critical for effective management of agroecosystems. The ArcGIS-based Agricultural Policy Environmental eXtender (ArcAPEX) model (version 1501) was employed to simulate streamflow, sediment, and nutrient yield in an agricultural watershed in northeastern Arkansas, and to evaluate scenario-based conservation practices. The ArcAPEX model was set up using topographical, soil, agronomical, and meteorological data. Measured daily data used for model calibration and validation included streamflow and sediment and nutrient yields derived from analysis of in-stream water samples collected from the Little River Ditches watershed in Northeastern Arkansas from 2015 to 2022. Sensitivity analysis, model calibration, and validation were conducted using the APEX auto-calibration and uncertainty estimator. The simulated streamflow and sediment, total phosphorus (TP), and total nitrogen (TN) yields were within the model evaluation criteria with higher reliability. The average annual streamflow and sediment, TP, and TN yields measured from the watershed were 667 mm, 3.39 tons/ha, 4.69 kg/ha, and 5.4 kg/ha and those from the ArcAPEX model simulations were 628 mm, 3.96 ton/ha, 5.41 kg/ha, and 5.69 kg/ha, respectively, during the study period. The scenario-based ArcAPEX model demonstrated that the combination of cover crops and filter strips reduced the runoff, sediment, and nutrient yields most effectively. Implementing cover crops and filter strips across 30% of the watershed reduced sediment yield by 26%, more than any other pollutant. This study validated ArcAPEX as an effective tool for simulating surface runoff and sediment and nutrient yields in agricultural watersheds and predicting the impact of conservation practices, demonstrating its value for complex agroecosystem management.

## Introduction

Agriculture is a cornerstone of Arkansas’s economy. In 2023, agriculture contributed approximately $24.3 billion, representing 14.5% of the state’s economy. Nationally, Arkansas ranked 14th in agricultural cash receipts ($14.5 billion in 2022) and 17th in crop production ($5.6 billion). Key crops included soybeans, valued $8 billion; rice, valued $1.68 billion; corn, valued $789 million; cotton, valued $612 million; wheat, valued $59 million; and peanuts, valued at $49 million (University of Arkansas Division of Agriculture Research and Extension, 2024; USDA ERS (U.S. Department of Agriculture Economic Research Service), 2024). Northeastern Arkansas counties, including Mississippi and Poinsett, are part of Arkansas’s Mississippi River Delta region and are dominated by major commodity crops, particularly soybeans, rice, cotton, and corn, which are well-suited to the region’s flat terrain, fertile soil, and irrigation systems. USDA National Agricultural Statistics Service data consistently show that these crops account for the majority of harvested acreage and farm income in Delta counties, reflecting the region’s role as one of the most productive agricultural areas in the United States (USDA NASS (United State Department of Agriculture, National Agricultural Statistics Service), 2024a, b, c, and d).

The Little River Ditches Watershed (LRDW) is a predominantly agricultural watershed of northeastern Arkansas in the Mississippi River Delta region and drains water from the cropping fields, transporting sediment and nutrient yield downstream ultimately to the Gulf via the Mississippi River (Reba et al., 2013). Robertson and Saad (2021) reported that agricultural activities contribute approximately 60% of nitrogen and 56% of phosphorus load to the Mississippi and Atchafalaya River Basin. Similarly, Ha et al. (2018) identified agriculture as the primary source of nitrogen and a major contributor of phosphorus in the Lower Mississippi River Basin (LRMB). Their study estimated annual rates of sediment, nitrogen, and phosphorus loadings from the LRMB to the Mississippi River at up to 33.9 ton/ha, 24 kg/ha, and 4.5 kg/ha, respectively.

To reduce pollutant yield from agricultural watersheds to freshwater river systems, cost-effective and ecologically sound conservation practices such as cover crops (USDA NRCS conservation Practice Code 340) and filter strips (USDA NRCS Conservation Practice Code 393) have been implemented (Thapa et al., 2024). Even though conservation practices such as cover crops and filter strips are beneficial to reduce pollutants, including sediment, nitrogen, and phosphorus yield from the watershed, direct monitoring of their benefits at the watershed level is often limited by economic and technical constraints. As a result, site-specific evaluations of conservation practices at the watershed scale in the LRDW have not yet been conducted. Process-based models like the Agricultural Policy Environmental eXtender (APEX) provide a practical alternative for estimating pollutant sources and evaluating conservation strategies when direct monitoring is not feasible (Thapa et al., 2025a). Compared to other process based model such as Soil and Water Assessment Tool (SWAT) or Environmental Policy Integrated Climate (EPIC) or Annualized Agricultural Non-Point Sources (AnnAGNPS), APEX model is superior for field or small scale watershed modeling since it has greater capability to incorporate detailed crop management practices, strong nutrient and carbon cycling, and is flexible in subarea and landscape modeling, which help to better reflect the actual streamflow, sediment, and nutrient yields from the agricultural watershed like LRDW (Golmohammadi et al., 2014; Pan et al., 2021; Senaviratne et al., 2018b; Taylor et al., 2015; Thapa et al., 2025a). Even though the ArcAPEX model- an APEX model implemented within the ArcGIS (Geographic Information System) interface- is very comprehensive, its widespread use has been limited due to its complexity, the extensive data requirements (such as weather, soil characteristics, crop management practices, and topography), and the large number of parameters involved in the simulation process (Feng et al., 2019; Nelson et al., 2024). APEX is a process based physical model, and its accuracy relies on the quality of input data that are based on the field or watershed and calibration parameters (Thapa et al., 2025a).

The goal of the study was to contribute to the ArcAPEX model parameters and performance within the APEX user community, as well as to simulate streamflow, sediment, and nutrient yield reduction resulting from the application of conservation practices in the LRDW. This study is particularly important because the ArcAPEX model previously applied at the field scale was limited to certain crops and management practices, which may not accurately reflect model parameterization at watershed scale with multiple crops and management practices in one of the most vital row crop-producing regions, the LMRB in the U.S. (Thapa et al., 2025a). Moreover, this study also used both long-term (8 years) and high-frequency datasets including daily streamflow data derived from 15-minute discharge measurements and daily sediment and nutrient yields estimated from weekly sediment and nutrient concentration data (Aryal & Reba, 2017; Thapa et al., 2024) for model development and validation and presented the scenario based agricultural conservation practices and its effect on the streamflow and sediment and nutrient yields reduction, which might be an alternative methodology to reduce time and cost required for collecting waters samples and analyzing them for sediment and nutrients to evaluate proposed conservation practices (Tuppad et al., 2010). To fulfill these research gaps, the study specifically aimed to (1) develop a calibrated ArcAPEX model and its parameters using daily measured streamflow, sediment, total phosphorus (TP) and total nitrogen (TN) yield at the outlet of the LRDW, (2) evaluate the effectiveness of the ArcAPEX model in simulating streamflow, sediment, and nutrient yield from the LRDW, and (3) assess reduction of pollutant yields due to implementation of scenario based conservation practices at varying areal coverages.

## Materials and Methods

### ArcAPEX model description

The APEX model (Steglich and Williams, 2008) is a process-based model written in FORTRAN. It has been modified to run on various applications, including Excel, known as APEXEditor (Leyton, 2019); ArcGIS, known as ArcAPEX (Tuppad et al., 2009); Windows, known as WinAPEX (Magre et al., 2006); open-source Quantum GIS known as QAPEX (Koo et al., 2022); and web-based decision making, known as Nutrient Tracking Tools (NTT; Saleh et al., 2011). During the APEX model setup and execution process, individual or combinations of APEX model variants can be used; however, combinations of APEX model variants are preferred since different APEX model variants have their unique capability and provide flexibility during model setup and execution. The APEX model implements physical, chemical, and biological processes for the prediction of hydrological and crop growth using weather, crop management, soil, and topographic data. APEX also includes routing components for hydraulic, sediment, nutrient, and pesticide movement between subareas, watershed outlets, groundwater in shallow aquifers, and conservation practices (Williams et al., 2008). In this study, we employed ArcAPEX model version 1501 since ArcAPEX has a GIS-based user interface that delineates the watershed boundary and subareas, and derives watershed characteristics, including topography, hydrology, land use, and soil spatial datasets. Additionally, the ArcAPEX model directly integrates the APEX parameters database that contains plant, tillage, fertilizer, pesticide, weather characteristics, and other information that are needed to simulate streamflow, nutrients, sediment, pesticides, crop yield, and economic budgeting in daily, monthly, and/or yearly time steps from a small watershed (Tuppad et al., 2009).

### Watershed location and characteristics

**Fig. 1:**
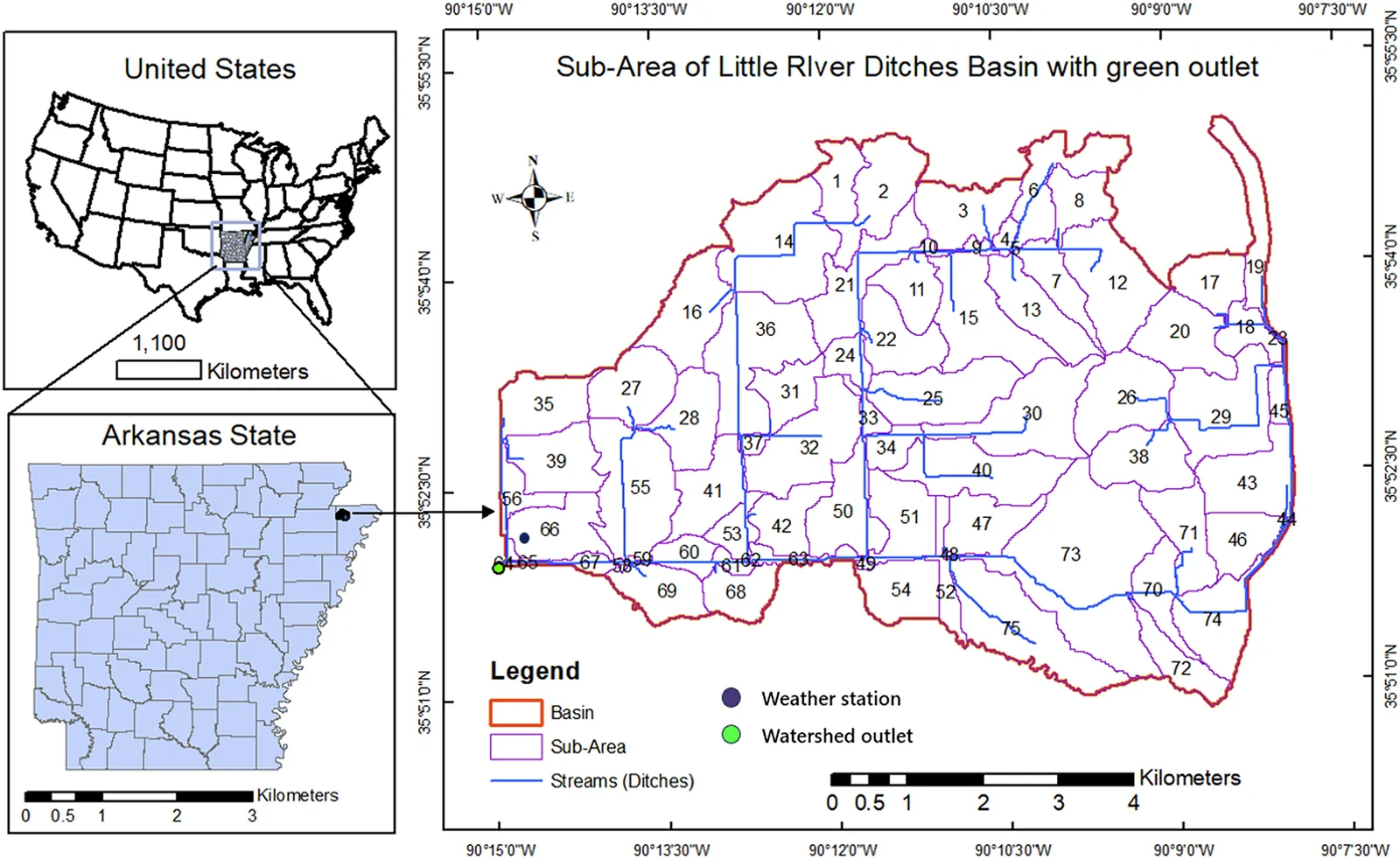
Location of the Little River Ditches Watershed with reference to the State of Arkansas and the USA

The study was conducted from 2015 to 2022, located in the LRDW in Arkansas, which is predominantly an agricultural watershed (Fig. 1). The LRDW covers approximately 53.78 square kilometers (13,289.3 acres). According to the United States Geological Survey, National Land Cover Database (2024) (United States Geological Survey, National Land Cover Database(NLCD) accessed 8 September 2024), over 87% of the land area consisted of row crops, primarily cotton (*Gossypium hirsutum* L.) and soybean (*Glycine max* [L.] Merr.) in rotation, while residential areas made up less than 11%. In addition to soybean and cotton, other crops such as peanuts (*Arachis hypogaea*), corn (*Zea mays* L.), sorghum (*Sorghum bicolor*), and watermelon (*Citrullus lanatus*) were also scattered within the study area (Dewitz, 2024).

According to the USDA Web Soil Survey (Soil Survey Staff, 2024; accessed 9 December 2024), approximately 62% of the study area is Routon-Dundee-Crevasse Complex (silt loam), 14.1% Tiptonville and Dubbs or Dundee silt loam, 7% Dundee-Dubbs-Crevasse complex, 6.1% Steele and Tunica soils, and 10.8% other soil types. The predominant soil hydraulic group is C/D (64.5%), followed by C (21.2%), A (12.4%), and D (1.8%). The area mainly employs conventional tillage practices, including field cultivators and disk bedders (Thapa et al., 2024). Most of the field is irrigated by furrow irrigation, and a few fields also use sprinkler irrigation utilizing shallow alluvial aquifers for irrigation (Reba and Massey, 2020). All fields are drained via surface field ditches rather than subsurface drains like tile drainage (Aryal et al., 2017). Fertilization in the fields follows the recommendations of the land-grant university based on soil tests (Thapa et al., 2024). Detailed information about the watershed can be found in Aryal and Reba (2017), and crop management practices can be found in Thapa et al. (2024) and the crop management and conservation practices section in this manuscript.

### Input data for APEX model

**Fig. 2:**
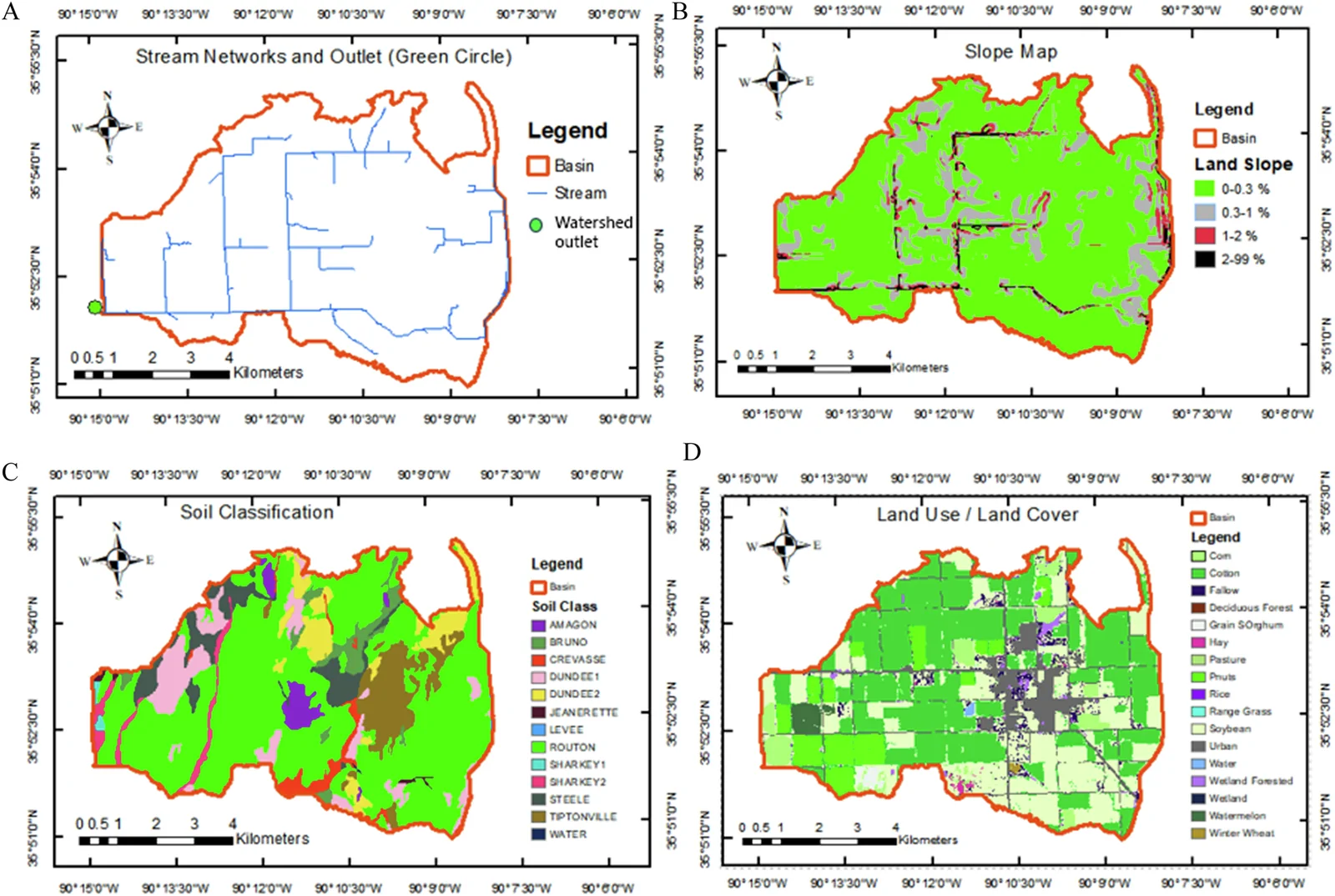
**A** Stream network (blue lines) with watershed outlet (green circle), **B** land slope, **C** soil class, and **D** land use/ land cover of the Little River Ditches Watershed, Arkansas

Generally, ArcAPEX models need subarea, soil, weather, and crop management inputs to run the model. The weather file contained maximum and minimum temperatures (°C), solar radiation (MJ/m²), average daily wind speed (m/s), precipitation (mm), and relative humidity (%) data recorded using a Campbell Scientific weather station (Campbell Scientific, Logan, UT) located inside the watershed. The weather station located within the watershed represented local weather conditions more accurately than a network of nearby stations, as spatially but sparsely distributed stations may not adequately capture localized weather phenomena such as rainfall amount and intensity. Rainfall characteristics are among the most influential factors affecting streamflow and pollutant yield in APEX model simulations. Therefore, we primarily used data from the watershed weather station, and we also found that most of the data collected from the weather station installed within the watershed were consistent with those from the nearby weather stations. We borrowed less than 2% of missing data from PRISM climate datasets (https://www.prism.oregonstate.edu/). The depth and velocity of flow in the drainage ditch or stream (at the green circle in the watershed, Fig. 2A) were measured continuously at 15-minute intervals using a pressure transducer (Teledyne Isco 2150 AV sensors) at the outlet of the watershed and a portable flowmeter (Marsh McBirney Inc. Flo-Mate 2000). Daily weather data were used using. DLY (file extension for daily weather input data) file and coded NGN = [2345] (NGN = the weather input code) to read all weather variables in the ArcAPEX control file while ArcAPEX model ran. The Soil Survey Geographical (SSURGO) Database was downloaded from the NRCS Web Soil Survey website (Fig. 2C; Soil Survey Staff, 2024). Crop management data were derived from the NLCD provided by the US Geological Survey (United States Geological Survey, 2024; accessed 8 September 2024; Fig. 2D). Composite water samples were collected each week using an automatic sampler (Teledyne Isco 6712, Lincoln, NE), and sediment, TP, and TN concentrations in the samples were measured at the Ecotoxicology Research Facility at Arkansas State University and the Delta Water Management Research Unit laboratories. The daily streamflow was calculated from the 15-minute interval field-collected data. Daily sediment, TP, and TN yield were estimated from the weekly sediment, TP, and TN concentrations from the water samples and daily discharge from the watershed. The compiled data were used for the model. The watershed-specific missing streamflow, sediment, TP, and TN yield data during measurement could not be borrowed from external sources. Consequently, these missing data were generated using the K-Nearest Neighbors (KNN; Chiu et al., 2019; Oktaviani and Putrada, 2022), a multivariate approach implemented in Python since many researchers have reported that the KNN imputation offers several methodological advantages over traditional imputation approaches such as mean, median, or regression-based imputation. First, KNN is a non-parametric method that does not require assumptions about the underlying data distribution, making it suitable for complex and nonlinear datasets. Second, it imputes missing values based on the similarity among observations, thereby preserving the natural structure and variability of the data more effectively than simple statistical replacement methods. In addition, KNN can handle both continuous and categorical variables and often produces more accurate estimates when strong relationships exist among variables, and is considered the most effective technique for hydrological and nutrient yield data imputation (Eishoeei et al., 2025; Malarvizhi & Thanamani, 2012; McRoberts, 2012; Thapa et al., 2025b). The few missing streamflow (<3%), sediment (<3.5%), TP (<4%), and TN (<4%) data were generated using a 5-nearest neighbor (KNN) based on Euclidean distance. The features used to fill missing streamflow, sediment, TP, and TN yields were maximum and minimum temperatures, solar radiation, average daily wind speed, precipitation, and relative humidity as predictors variables (Thapa et al., 2025a).

### ArcAPEX model setup

The APEX1501 extension in ArcGIS version 10.8 was employed to simulate the streamflow, sediment, and nutrient yield from the LRDW. The inputs used for the ArcAPEX model were topography, soil, weather, land use/land cover, and crop management data. The watershed and subarea were delineated using the 10 m digital elevation model (DEM), and the stream network was defined using masking and burn-in functions in the ArcAPEX model. During the subarea delineation process, we aimed to increase the number of subareas as much as possible to allow us to manipulate different crop management practices for each subarea. In this study, 75 subareas were created (Fig. 1), and the subarea parameters, including land management, soil type, slope, and channel routing information etc. were calculated from the delineated subareas. The weather and crop management practices data were added subsequently during the model setup. The ArcAPEX model was run for crop management practices implemented in LRDW from 2015 to 2022. After completing the APEX model run, the model was calibrated and validated using daily measured streamflow, sediment, TP, and TN yield. After calibrating and validating the model, a scenario-based study was conducted to assess the impact of areal coverage of cover crops and filter strips on reducing streamflow, sediment, and nutrient yield from the watershed. These conservation practices are widely adopted by farmers to mitigate pollutant runoff, while also helping to retain sediment and nutrients on the field, promoting sustainable crop production and soil health.

### Crop management and conservation practices

The watershed was primarily planted with soybean and cotton in rotation. Some fields were occasionally planted with peanuts, corn, sorghum, and watermelon. The APEX model simulation used crop management based on the producer’s conventional crop management practices. Conventional tillage practices such as disk bedders are used to reshape the bed in the fall, and cultivators are used to flatten the bed before planting. Before planting, a third of the recommended dose of nitrogen-based fertilizer and the dose of phosphorus- and potassium-based fertilizers were applied in the field (Table 1). The rest of the nitrogen-based fertilizer was applied two times in the mid-season. The crops were planted using regular planters (PLRG12RW in APEX Database) that have 12 rows and can plant 9 seeds/m^2^ in 40 mm deep. The irrigation started after clearing the furrow with the row cultivator/ lister. The typical irrigation amount and date depend on crop water demand and weather conditions. However, the amount and timing of fertilization vary based on the individual field’s soil test results. For simplicity, the fertilization rate, seed rate, and irrigation amounts used are kept constant for simulation purposes per crop (Table 1). Winter wheat (*Triticum aestivum*) and rye grass (*Secale cereale*) are typically seeded at a rate of 350 seeds m^-^² as a cover crop after harvesting the main crop and is terminated 2 to 3 weeks before planting the main crops (Thapa et al., 2024; Thapa et al., 2025a). Similarly, the seed rate, irrigation amount, planting and harvesting times were applied on the same date for all fields based on the crop type and year for generalization purposes in the watershed. The details of the crop planting year and subarea based on the NLCD and ArcAPEX output file are shown in Table 2.

**Table 1:** Fertilization, seed rate, and irrigation amount used in crop management section of ArcAPEX model

Crop/ Management	Cotton	Soybean	Corn	Sorghum	Peanuts	Watermelon
N-Fertilizer 3 times (Urea kg/ha)	55	-	85	67	-	45
P-Fertilizer (DAP kg/ha)	83	45	100	55	40	100
K-Fertilizer (Muriate of potash kg/ha)	67	67	100	78	60	100
Planting (seeds/m²)	9	32	8.5	18.5	15	0.25
Irrigation (mm X number of applications)	76.2 × 5	51 × 5	90 × 5	80 × 6	80 × 6	50 × 6

(Sources: Local producer report, Aryal and Reba (2017), Reba et al. (2020), Thapa et al. (2024), Espinoza and Ross (2008), and Tacker et al. (2003))

**Table 2:** Crops planted in the subareas of the Little River Ditches Watershed, Arkansas, in the ArcAPEX model from 2015 to 2022 based on the dominant crops obtained from National Land Cover Data (NLCD year) (WW= Winter wheat cover crop)

Year	Subarea												
	4, 5, 6, 12, 28	9, 23, 44, 70, 74	1, 2, 21, 25	3, 14, 26, 29, 31, 32, 36, 43	7, 48, 51	8	10	11, 15	13, 18, 63	16, 47	17, 20	19, 37, 38	22
2015	Cotton	Soybean	Soybean	Soybean	Soybean	Soybean	Soybean	Cotton	Soybean	Soybean	Cotton	Soybean	Soybean
2016	Cotton	Soybean	Cotton	Cotton	Cotton	Soybean	Soybean	Cotton	Cotton	Cotton	Cotton	Soybean	Cotton
2017	Cotton	Soybean	Cotton	Cotton	Soybean	Soybean	Peanuts	Cotton	Peanuts	Soybean	Cotton	Cotton	Soybean
2018	Cotton	Soybean	Peanuts	Cotton	Cotton	Soybean	Cotton	Cotton	Cotton	Cotton	Cotton	Cotton	Cotton
2019	Cotton	Soybean	Cotton	Cotton	Cotton	Cotton	Cotton	Cotton	Cotton	Cotton	Peanuts	Cotton	Corn
2020	Cotton	Soybean	Cotton	Cotton	Cotton	Corn	Cotton	Peanuts	Cotton	Peanuts	Cotton	Cotton	Cotton
2021	Cotton	Soybean	Cotton	Cotton	Cotton	Cotton	Cotton	Cotton	Peanuts	Cotton	Cotton	Cotton	Peanuts
2022	Cotton	Soybean	Cotton	Cotton	Cotton	Cotton	Cotton	Cotton	Cotton	Cotton	Peanuts	Cotton	Cotton
Year/ Subarea	24	27	30, 40	33	34	35	39	41,53,55, 59, 60, 67	42	45	46, 57, 72, 73	49	
2015	Peanuts	Soybean	Urban	Cotton	Cotton	Soybean	WW/ Soybean	Soybean	Soybean	Soybean	Soybean	WW/ Soybean	
2016	Cotton	Peanuts	Urban	Cotton	Cotton	Soybean	Watermelon	Peanuts	Cotton	Cotton	Cotton	WW/ Soybean	
2017	Peanuts	Soybean	Urban	Cotton	Cotton	Peanuts	Cotton	Cotton	Peanuts	Cotton	Soybean	WW/ Soybean	
2018	Cotton	Cotton	Urban	Corn	Cotton	Cotton	Cotton	Cotton	Cotton	Cotton	Soybean	WW/ Soybean	
2019	Cotton	Peanuts	Urban	Cotton	Cotton	Cotton	Peanuts	Peanuts	Cotton	Peanuts	Cotton	WW/ Soybean	
2020	Peanuts	Cotton	Urban	Cotton	Cotton	Peanuts	Cotton	Cotton	Cotton	Cotton	Cotton	WW/ Soybean	
2021	Cotton	Cotton	Urban	Cotton	Peanuts	Cotton	Cotton	Cotton	Cotton	Cotton	Cotton	WW/ Soybean	
2022	Cotton	Cotton	Urban	Cotton	Cotton	Cotton	Cotton	Peanuts	Cotton	Peanuts	Soybean	WW/ Soybean	
Year/ Subarea	50	52, 54	56	58	61	62	64	65, 66	68	69	71	75	
2015	Cotton	Cotton	WW/ Soybean	Fallow	Soybean	Soybean	forest	Cotton	Soybean	Soybean	Soybean	WW/ Soybean	
2016	Cotton	Soybean	Corn	Fallow	Soybean	Soybean	forest	Corn	Sorghum	Peanuts	Cotton	Soybean	
2017	Cotton	Peanuts	Cotton	Fallow	Cotton	Soybean	forest	Cotton	Cotton	Cotton	Soybean	Soybean	
2018	Cotton	Cotton	Cotton	Fallow	Soybean	Soybean	forest	Cotton	Soybean	Corn	Cotton	Soybean	
2019	Cotton	Cotton	Corn	Fallow	Peanuts	Cotton	forest	Corn	Peanuts	Peanuts	Soybean	Cotton	
2020	Cotton	Cotton	Cotton	Fallow	Cotton	Cotton	forest	Cotton	Cotton	Cotton	Cotton	Soybean	
2021	Cotton	Cotton	Corn	Fallow	Soybean	Soybean	forest	Cotton	Soybean	Cotton	Cotton	Cotton	
2022	Peanuts	Cotton	Cotton	Fallow	Cotton	Cotton	forest	Cotton	Cotton	Peanuts	Cotton	Soybean	

Nine scenario-based cover crops and filter strip conservation practices (3 for cover crops, 3 for filter strips, and 3 for a combination of cover crops and filter strips) were implemented on 10%, 20%, and 30% of the watershed area. However, occasionally the watershed was planted with winter wheat as cover crops in a negligible portion of the watershed (Table 2). This approach reflects the practical implementation of these conservation practices on farmland within the watershed. The scenario-based cover crops and filter strips in the watershed subareas were assigned randomly; however, we chose the subareas that produced average runoff and sediment yield rather than extremely high or low runoff or sediment yield to avoid over- or underestimation. The selected subarea numbers 4, 19, 21, 43, 46, 53, 56, and 74 covered almost 10% area of the watershed, subarea numbers 4, 18, 19, 21, 22, 24, 33, 36, 43, 46, 50, 53, 56, 60, 70, and 74 covered almost 20% area of watershed, and subarea number 4, 18, 19, 21, 22, 24, 25, 27, 31, 32, 33, 36, 42, 43, 46, 50, 53, 56, 60, 68, 70, and 74 covered almost 30% area of the watershed area. For the cover crop scenario, the selected subareas were planted with rye (Secale cereale) grass as a cover crop. The filter strip conservation practice was also applied both separately and in combination with cover crops in the same subareas. The filter strip parameters were set in a subarea file based on guidelines provided in the APEX parameterization manual for conservation practices (Steglich, 2016) and recommended by Senaviratne et al. (2018b). The parameters used for filter strips set up in the subarea file were erosion control practice factor (PEC) of 0.6, average upland slope (SLP) of 0.25* slope of the field, average upland slope length (SPLG) of 20 meters, Manning’s N for upland (UPN) of 0.15, reach floodplain length (RFPL) to 0.02 kilometers, fraction of floodplain flow (FFQP) of 0.95. Similarly, the land use number (LUN) 22 for Kentucky 31 tall fescue grass [*Lolium arundinaceum* (Schreb.) Darbyish] (Senaviratne et al., 2018a) was used in the operation schedule file.

### ArcAPEX model calibration, validation, and evaluation

The APEX model was warmed up for 10 years and calibrated and validated for streamflow and sediment, TP, and TN yield using daily data from 2015 to 2019 and 2020 to 2022, respectively. The daily simulated streamflow (WYLD) and sediment (MUSS), TP (QP), and TN (QN) yield from the watershed were taken from the daily watershed outlet (.DWS) file. Initially, sensitivity analysis, followed by calibration and validation, was conducted using APEX-CUTE, the Dynamically Dimensioned Search (DDS) algorithm widely used for APEX model calibration (Kamruzzaman et al., 2020; Thapa et al., 2025a), following recommendations from the APEX-CUTE manual (Wang et al., 2014), then fine-tuned manually (Bosch et al., 2020; Kamruzzaman et al., 2020). This study performed the calibration and validation processes sequentially, starting with streamflow and ending with sediment, TP, and TN yield.

During the calibration process, all parameters were selected for water balance and streamflow using APEX-CUTE (Wang et al., 2014), including peak streamflow rate (APM), maximum groundwater storage (GWSo), groundwater residence time (RFTo), return flow (return flow plus deep percolation; RFPo), and saturated conductivity adjustment factor (SATO) (Table 3). Similarly, all parameters were selected for the soil erosion/sediment, TN, and TP calibration process. The most sensitive parameters (influential parameters in calibration) were also manually adjusted based on literature and professional judgment (Baffaut et al., 2017; Senaviratne et al., 2018a).

**Table 3:** ArcAPEX model performance evaluation criteria

Parameter	R²	NSE	PBIAS	RSR
Daily surface streamflow	≥0.7	≥0.5	≤±20%	≤0.70
Daily sediment yield	≥0.6	≥0.45	≤±25%	≤0.70
Daily TP yield	≥0.5	≥0.45	≤±30%	≤0.75
Daily TN yield	≥0.5	≥0.45	≤±30%	≤0.75

*NSE* Nash-Sutcliffe efficiency, *PBIAS* Percent bias, *R²* Coefficient of determination, *RSR* RMSE observation standard deviation ratio, *TP* Total Phosphorus, *TN* Total Nitrogen

The performance of the APEX model was evaluated by comparing observed values with predicted or simulated values using various statistical measures. The statistical parameters used for model performance evaluation included the coefficient of determination (R²; Eq. 1) (Senaviratne et al., 2018a), root mean square error (RMSE; Eq. 2) (Thapa et al., 2025a), Nash-Sutcliffe efficiency (NSE; Eq. 3) (Nash and Sutcliffe, 1970), percent bias (PBIAS; Eq. 4) (Gupta et al., 1999), and RMSE-observation standard deviation ratio (RSR; Eq. 5) (Thapa et al., 2025b). The performance evaluation criteria and satisfactory level for the ArcAPEX model were established based on recommendations by Bosch et al (2020) and Moriasi et al. (2015) and summarized in Table 3. Higher R^2^ and NSE values and lower RMSE, PBIAS, and RSR values indicate better model predictability (Thapa et al., 2025a). The average annual measured and simulated streamflow, sediment, TP, and TN yields were also compared using a paired t-test at the 95% significance level, and the corresponding *p*-values were reported. Model performance was also evaluated using estimation error, uncertainty interval, and reliability. When a manual parameter adjustment resulted in improved model performance, the parameter value was retained; adjustments were stopped once further changes led to a decline in performance. In some cases, the model was calibrated by adjusting multiple parameters simultaneously, followed by an evaluation of overall model performance. Once calibration and validation were completed for the baseline (existing) conditions, the same APEX model was used for scenario-based conservation practices that were applied during the simulation process.

1 $${R}^{2}={\left[\frac{\mathop{\sum }\nolimits_{i=1}^{n}\left({O}_{i}-\bar{O}\right)\left({S}_{i}-\bar{S}\right)}{\mathop{\sum }\nolimits_{i=1}^{n}{({O}_{i}-\bar{O})}^{2}\mathop{\sum }\nolimits_{i=1}^{n}{({S}_{i}-\bar{S})}^{2}}\right]}^{2}$$

2 $${RMSE}=\sqrt{\frac{\,{\mathop{\sum }\nolimits_{i=1}^{n}({O}_{i}-{S}_{i})^{2}}\,}{n}}$$

3 $${NSE}=1-\left[\frac{\mathop{\sum }\nolimits_{i=1}^{n}{({O}_{i}-{S}_{i})}^{2}}{\mathop{\sum }\nolimits_{i=1}^{n}{({O}_{i}-\bar{O})}^{2}}\right]$$

4 $${PBIAS}=\frac{\mathop{\sum }\nolimits_{i=1}^{n}\left({O}_{i}-{S}_{i}\right)}{\mathop{\sum }\nolimits_{i=1}^{n}{O}_{i}}\,\times \,100\, \%$$

5 $${RSR}=\,\frac{{RMSE}}{{{STDEV}}_{{Obs}}}\,=\sqrt{\frac{\,{\mathop{\sum }\nolimits_{i=1}^{n}({O}_{i}-{S}_{i})^{2}}\,}{{\mathop{\sum }\nolimits_{i=1}^{n}\left({O}_{i}-\bar{O}\right)}^{2}}}$$

Where ${o}_{i}$ and ${s}_{i}\,$ are the observation and simulated values, respectively; ō is the mean of the observation data, and n is the total number of observations.

### Model uncertainty and reliability analysis

Since hydrological models exhibit uncertainty in parameters and response variables (Maskey et al., 2025), a comprehensive quantitative assessment of uncertainty and reliability in predicting streamflow, sediment, and nutrient yield was conducted to estimate potential sources of error in the ArcAPEX model prediction. For the uncertainty analysis, we employed prediction error and uncertainty interval methods using daily measured and predicted data from 2015-2022. In the uncertainty analysis, the estimation error ($${e}_{i}$$) for each day was calculated (Eq. 6), and the average estimation error ($${e}_{{avg}}$$; Eq. 7) was calculated. The standard deviation ($${{sd}}_{e})$$ of the estimation error was calculated using daily estimation error and average estimation error (Eq. 8). The lower and upper confidence interval ($${{CL}}^{\pm }$$) was calculated using average estimation error and standard deviation (Eq. 9).

6 $${e}_{i}={measured}\,{value}-{predicted}\,{value}$$

7 $${e}_{{avg}}=\mathop{\sum }\limits_{i=0}^{n}\frac{{e}_{i}}{n}$$

8 $${{sd}}_{e}=\,\sqrt{\frac{\mathop{\sum }\nolimits_{i=1}^{n}{({e}_{i}-{e}_{{avg}})}^{2}}{n-1}}$$

9 $${{CL}}^{\pm }={e}_{{avg}}\,\pm \,Z{{Sd}}_{e}$$

Where Z is standard normal value corresponding to the 95% confidence level (Devore, 1995). The width of the confidence band was determined by subtracting the lower confidence limit from the upper confidence limit (Kumar et al., 2022). Similarly, the uncertainty interval (U95) was estimated using the following equation:

10 $$U95=\left(\frac{1.96}{n}\right)\sqrt{{\sum }_{i=1}^{n}{\left({O}_{i}-{\bar{O}_{i}}\right)}^{2}+{\sum }_{i=1}^{n}{\left({O}_{i}-{S}_{i}\right)}^{2}}$$

Where ${S}_{i}$ is predicted values, and ${O}_{i}$ is measured values, and $\bar{{O}_{i}}\,$ is the mean of measured values (Saberi-Movahed et al., 2020).

Reliability analysis is a statistical method that shows the consistency of model performance under different conditions (Saberi-Movahed et al., 2020). Reliability is calculated as:

11 $${Reliability}=\left(\frac{100 \% }{n}\right)\mathop{\sum }\limits_{i}^{n}{K}_{i}$$

where K_i_ is obtained by a two-step process. First, the relative average error (RAE) of *i*th component is calculated by using the percentage of absolute value of estimation error (Eq. 12).

12 $${{RAE}}_{i}=\left|\frac{{O}_{i}-{S}_{i}}{{O}_{i}}\right|\times 100 \%$$

where, ${s}_{i}$ is predicted value and ${o}_{i}$ is measured values.

Second, the value of K_i_ is determined based on a threshold Δ value. If RAE_i_ ≤ Δ, then K_i_ = 1; otherwise, K_i_ = 0. The threshold Δ value is typically set to 0.2 or 20% based on the Chinese Standard (Saberi-Movahed et al., 2020).

## Results and discussion

### Sensitive and calibration parameters

For the APEX model sensitivity analysis and calibration process, APEX-CUTE (Wang et al., 2014) was utilized. APEX-CUTE identified the most sensitive parameters: parameter 92 - runoff volume adjustment coefficient, which is inversely related to daily CN adjustment, was most sensitive for runoff; parameter 19 - sediment routing coefficient for sediment load; parameter 59 - P upward movement by evaporation coefficient for TP load; and parameter 14 - nitrate leaching ratio for TN load from the watershed. Following the sensitivity analysis, the APEX model was calibrated and validated using APEX-CUTE. During calibration, the hydrologic budget with sediment and nutrient transport was considered. Therefore, despite using APEX-CUTE, manual calibration was performed utilizing professional judgment and literature values to refine sensitive parameters thereby optimizing statistical metrics (maximum R^2^ and NSE, minimum RMSE, PBIAS, and RSR). In this study, some parameter values obtained during the calibration process were outside the recommended range suggested in the APEX manual; similar findings were also reported by Mason et al. (2020) and Senaviratne et al. (2018a). The most influential calibrated model parameters are presented in Table 4.

**Table 4:** APEX model key parameters range and calibrated value for Little River Ditches Watershed

Parameter	Description	Range	Calibrated value
Water balance			
APM	Peak runoff rate – rainfall energy adjustment factor	0–1	0.82
GWSo	Maximum ground water storage in mm	5–200	109.95
RFTo	Ground water residence time in days	0–365	33.43
RFPo	Return flow/ (return flow+ deep percolation)	0–1	0.64
SATo	Saturated conductivity adjustment factor	0.01–10	2.27
5	Soil water lower limit	0–1	0.378
12	Soil evaporation coefficient	1.5–2.5	2.1355
15	Runoff CN Residue Adjustment Parameter	0–0.3	0.15
16	Expands CN retention parameter	1–1.5	1
17	Soil evaporation – plant cover factor	0–0.5	0.25
20	Runoff curve number initial abstraction	0.05–0.4	0.4
23	Hargreaves PET equation coefficient	0.0023–0.0032	0.0032
25	Exponential coefficient used to account for rainfall intensity on curve number	0–2	2.45*
34	Hargreaves PET equation, exponent	0–0.6	0.6
40	Groundwater storage threshold	0.001–1	0.491
42	SCS curve number index coefficient	0.3–2.5	1.95
49	Maximum rainfall intercepted by plant canopy	0–15	2.038
50	Rainfall interception coefficient	0.05–0.3	0.183
51	Water stored in litter	0.1–0.9	0.688
61	Soil water upward flow limit	0.05–0.95	0.126
91	Flood evaporation limit	0.001-1	0.004
92	Runoff volume adjustment for direct link, inversely related to runoff	0.1–2	0.924
Soil erosion			
13	Wind erodibility coefficient	0–3	1.9
18	Sediment routing exponent	1–2	1.41
19	Sediment routing coefficient	0.0001–0.05	0.04
45	Sediment routing travel time coefficient	0.5–10	5
69	Coefficient adjustment microbial activity function in the topsoil	0.1–1	0.98
70	Microbial decay rate coefficient	0.5–1.5	1
P processes			
8	Soluble phosphorus runoff coefficient	10–20	20
32	Organic P loss exponent	1–1.2	1
57	P enrichment ratio coefficient for routing	0.05-2	0.05
59	P upward movement by evaporation coefficient	1–20	17
84	Coefficient regulating P flux between labile and active pool	0.0001–0.001	0.001
96	Soluble Phosphorus Leaching KD value	1–15	1
N processes			
4	Water storage N leaching	0–1	0.85
14	Nitrate leaching ratio	0.1–1	0.333
29	Biological mixing efficiency	0.0001–0.01	0.5*
31	Maximum depth for biological mixing	0.1–0.3	0.3
36	Upper Limit of Daily Denitrification rate	0.0001–0.5	0.4
72	Volatilization/nitrification partitioning coefficient	0.05–0.5	0.145
86	Nitrogen and Salt Upward movement by evaporation coefficient	0.001–20	0.005

(*Calibrated parameters are outside the recommended range in the APEX manual.)

### Calibration and validation results

**Fig. 3:**
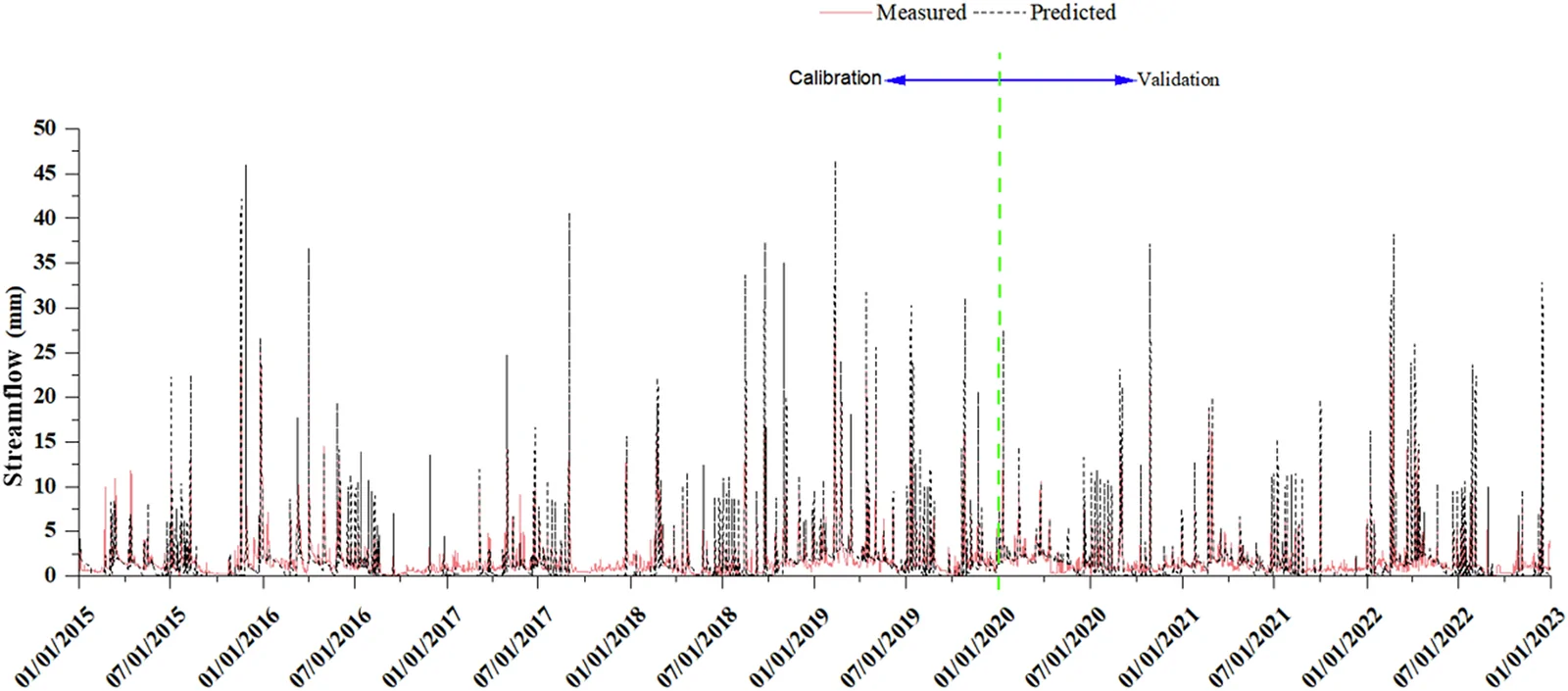
Measured and ArcAPEX model simulated daily streamflow hydrograph from the Little River Ditches watershed during calibration and validation periods

**Fig. 4:**
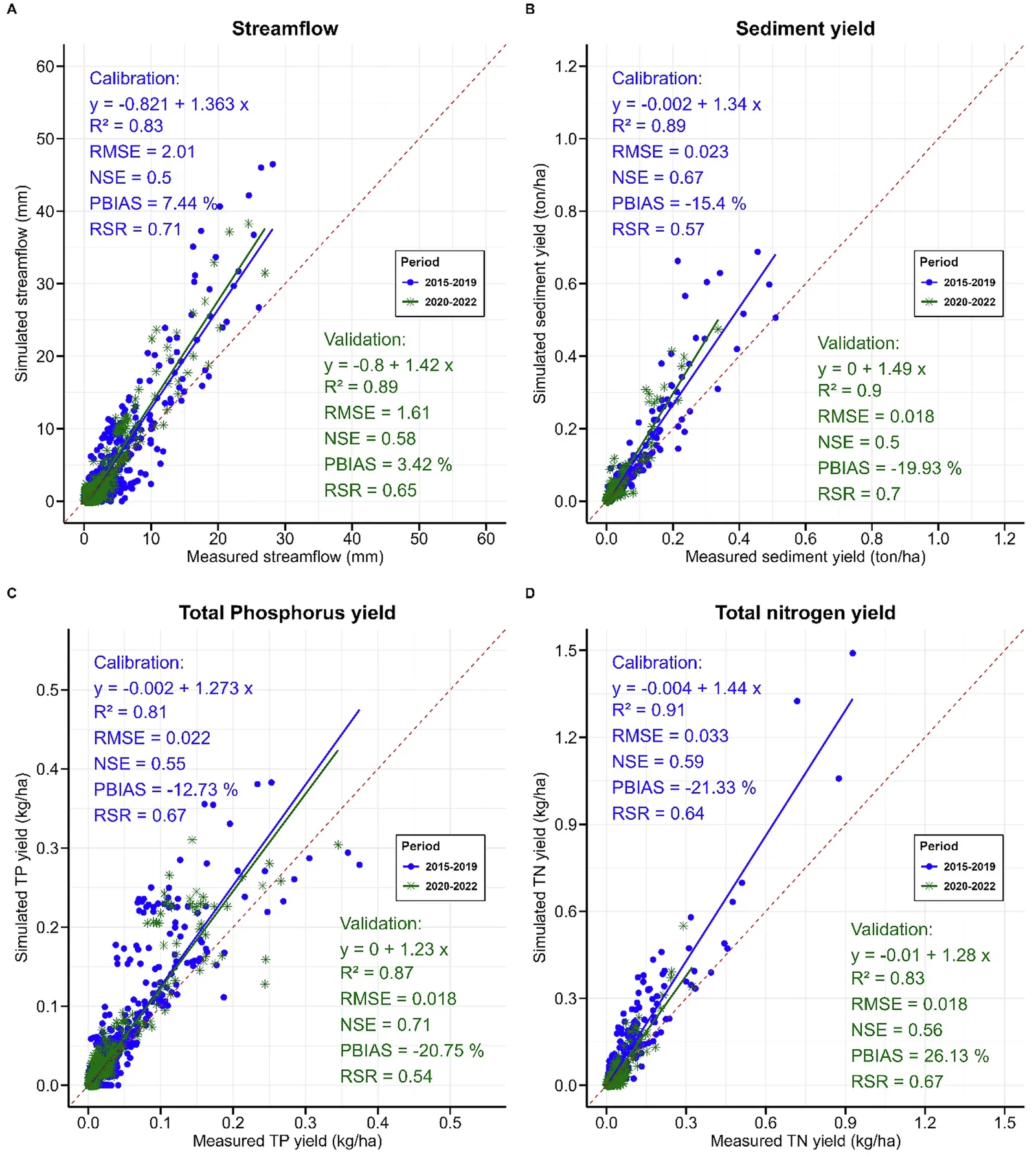
Scatter plot of daily measured and APEX model simulated streamflow (**A**), sediment (**B**), total phosphorus (**C**), and total nitrogen (**D**) yields from the Little River Ditches watershed. The blue dots and lines are for the calibration period (2015–2019), and green asterisks and lines are for the validation period (2020–2022). The red dashed line is 1:1 (45°) line and represents perfect agreement between measured and simulated value perfectly

**Fig. 5:**
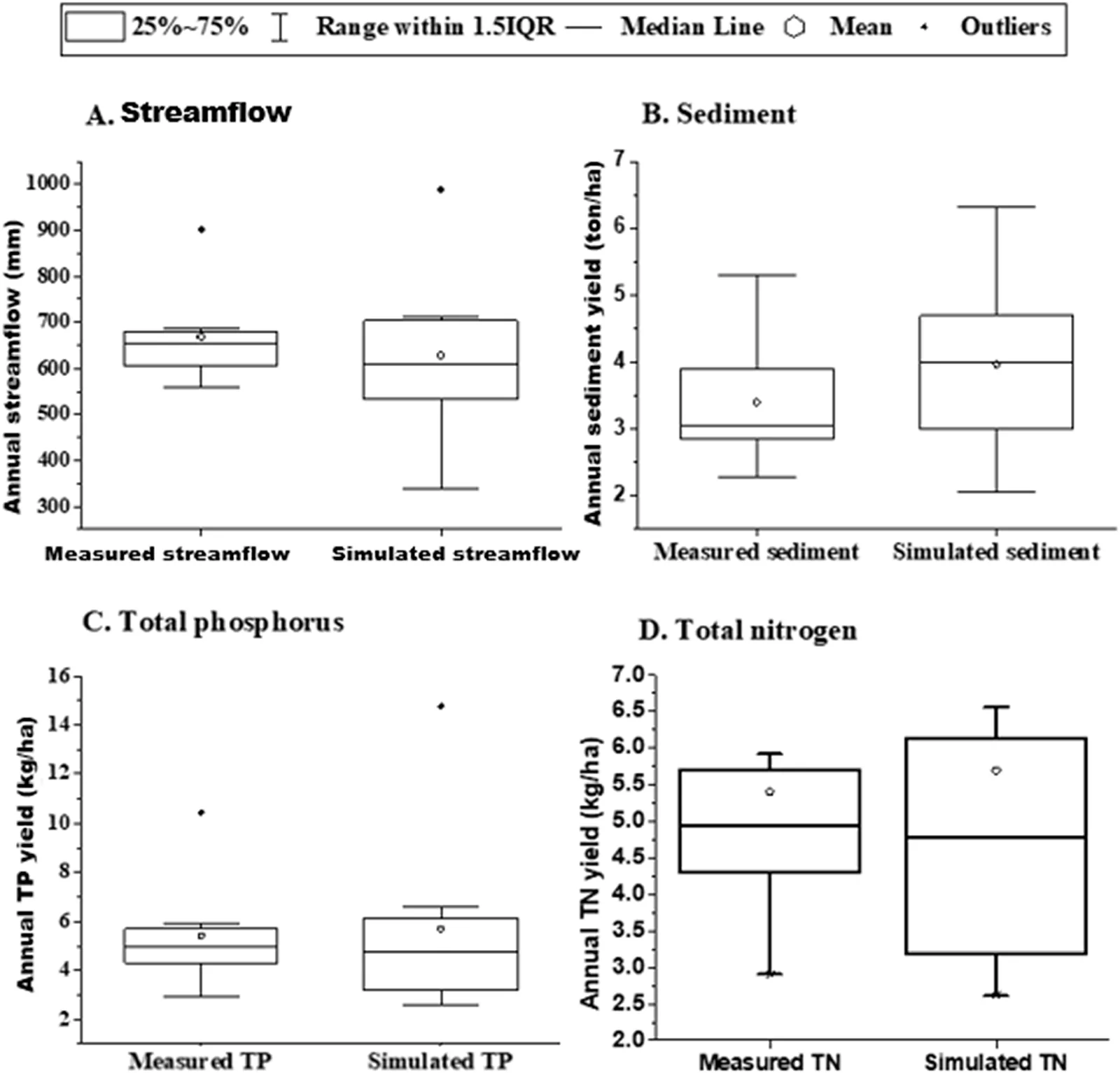
Measured and APEX model simulated annual streamflow (**A**) and sediment (**B**), total phosphorus (**C**), and total nitrogen (**D**) yields from the Little River Ditches watershed using data from 2015 to 2022

The calibrated ArcAPEX model’s simulated daily streamflow closely matched the measured streamflow (Fig. 3), with R^2^, RMSE, NSE, PBIAS, and RSR values of 0.83, 2.01, 0.5, 7.44%, and 0.71, respectively, during calibration, and 0.89, 1.61, 0.58, 3.42%, and 0.65, respectively, during validation (Fig. 4A). Similar results were reported by Thapa et al. (2025a) who found R^2^, RMSE, NSE, PBIAS, and RSR values to be 0.75, 2.40, 0.61, 21.92%, and 0.62, respectively, during streamflow validation in control field, and 0.72, 1.62, 0.57, 4.95%, and 0.66, respectively, during streamflow validation in the treatment field in the field-scale study in the LRDW. The simulated streamflow result also showed higher R^2^, NSE, and lower RMSE, PBIAS, and RSR during validation than during calibration, which is attributed to differences in data variability and hydrological conditions between the two periods. The average annual rainfall during the study period was 1556 mm, ranging from 1238 mm in 2017 to 2014 mm in 2019. Corresponding to this rainfall, the measured annual average streamflow from the watershed was 668 mm, while the ArcAPEX model simulated average annual streamflow was 628 mm, with no significant differences between them (*p* = 0.3, paired t-test); Fig. 5A). The field located within this watershed had an average streamflow of 461 mm per hectare, which accounted for approximately 27.13% of the rainfall and 9.2% of the irrigation (Thapa et al., 2025a). Although this study reported a higher amount of streamflow compared to the field-scale study, irrigation within the watershed varied depending on crop type and weather conditions during the cropping season as well as land cover/ land use of the watershed. Overall, the present study shows that less than 40% of the rainfall was converted to streamflow from the watershed, which is higher than the 30% runoff reported by Bosch et al. (2020). The higher percentage of streamflow in LRDW compared to the field located near Tifton, Georgia, reported by Bosch et al. (2020), may be due to spatiotemporal, hydrogeological, and crop management practice variability. The ArcAPEX model, which uses a hydrological process based on the water balance equation (Arnold et al., 1998), requires measurement of various hydrological processes, including percolation, return flow, lateral flow, and evapotranspiration to ensure accurate simulation. Although not all hydrologic processes were measured during the study, the streamflow measured daily at the outlet from the watershed matched the ArcAPEX model output by adjusting different hydrology-related parameters.

**Fig. 6:**
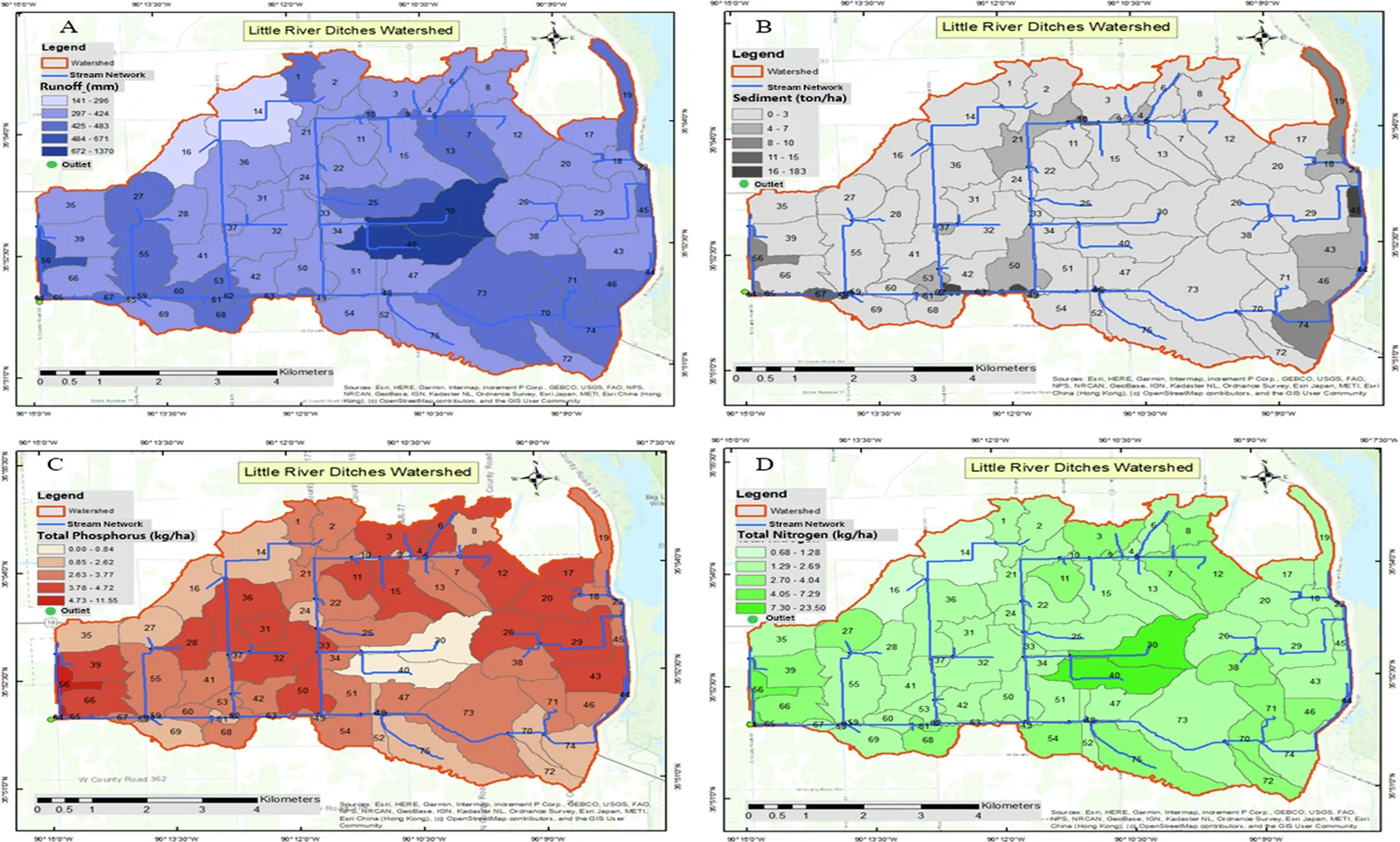
**A** Simulated annual streamflow (mm), **B** sediment (tons/ha), **C** total phosphorus (kg/ha), and **D** total nitrogen (kg/ha) yields from the Little River Ditches watershed

Simulated runoff across the various subareas, as depicted in Fig. 6A, revealed a spatial pattern in which subareas adjacent to the stream exhibited notably higher runoff. This trend is likely influenced by the proximity to the stream and the presence of steeper slopes, which enhance surface water conveyance and reduce infiltration opportunities (Figs. 2B and 6A). Other subareas with high runoff included subareas 30 and 40. A comparison of Figs. 2D and 6A evidently shows that subareas 30 and 40 are predominantly urban, in contrast to the more agriculturally dominated subareas. Urban areas typically generate increased runoff where impervious surfaces limit infiltration and vegetation uptake, thereby accelerating surface runoff (Dunne et al., 1991; Xu et al., 2020).

The R^2^, RMSE, NSE, PBIAS, and RSR values were 0.89, 0.023, 0.67, -15.4%, and 0.57, respectively, during the calibration period, while the same parameters were 0.9, 0.018, 0.5, -19.93%, and 0.7, respectively, during the validation period for the daily sediment yield (Fig. 4B). The average annual measured sediment yields were similar to the average annual simulated sediment yield for the study period (Fig. 4B). However, there was significant difference (p = 0.04, paired t-test) between the measured (3.39 tons/ha) and simulated average annual sediment (3.97 tons/ha) yields from the watershed. The average annual sediment yield from the LRDW was comparable to the 6.66 tons/ha sediment load reported by McConnell et al. (2005) for conventional tillage cotton fields in southeastern Arkansas. Similarly, Senaviratne et al. (2018a) reported local calibrated, remote calibrated, remote partially calibrated, and best judgment APEX simulated sediment load results from with and without six conservation practice scenarios and reported sediment load from 2.4 to 7.2 tons/ha from watersheds 2.69 to 31.7 ha in Missouri, which is similar to the results obtained in this study. Sharpley et al. (2020) reported up to 1.65 tons/ha from hay fields in the Big Creek Watershed, Arkansas, and Endale et al. (2014) reported 1.82 tons/ha from cotton and peanut fields in Tift County, Georgia, while Bosch et al. (2020) simulated 1.5 tons/ha sediment yield from the same field in Georgia using APEX. These values are lower than the sediment yield measured in this study, likely due to spatiotemporal and management differences. The sediment load reported by Sharpley et al. (2020) was from fields with buffers, and Endale et al. (2014) reported from various sloping lands, which were associated with different crop management practices compared to this study.

The ArcAPEX simulated results indicated that the subarea located near the main stream (routing reach) generated a higher sediment yield compared to subareas farther from the stream (Fig. 6B). The elevated sediment yield is likely due to a combination of steeper slopes (Fig. 2B), greater runoff, and shorter transport distances. Even though the subareas 30 and 40, primarily urbanized (Fig. 2D), produced higher runoff, it did not contribute significantly to the sediment load. This is likely due to the prevalence of impervious surfaces such as concrete and pavement, which limit soil exposure and erosion potential, thereby reducing sediment generation.

The APEX simulated TP yield was within a satisfactory level of performance evaluation criteria. During the calibration period, the R^2^ value was 0.81, RMSE value was 0.022, NSE value was 0.55, PBIAS value was −12.73%, and the RSR value was 0.67 (Fig. 4C). During the validation period, the R^2^ value was 0.87, RMSE value was 0.018, NSE value was 0.71, PBIAS value was -20.75%, and the RSR value was 0.54. The average annual measured TP yield was 4.69 kg/ha, whereas the simulated average annual TP yield was 5.41 kg/ha (Fig. 5C). There was significant difference (*p* = 0.004, paired t-test) between them. The significant difference between the annual average measured and simulated TP yields may be attributed to the strong correlation between sediment yield and TP yield (Thapa et al., 2024; Thapa et al., 2025a). In this case, the annual measured and simulated sediment yields were also significantly different, although the significance level was relatively low (p = 0.04), which may have influenced the significance level of annual average measured and simulated TP yield. However, the measured TP yield in this study was within the range of 0.03 to 5 kg/ha reported by Sharpley et al. (2020) in hay pasture fields in the Big Creek Watershed in Arkansas. However, Osmond et al. (2017) reported a TP yield of 2.12 kg/ha/yr from the rotational grazing and litter field in northwest Arkansas and 5.05 kg/ha/yr to 19.8 kg/ha/yr from central Georgia. Compared to the results from this study, the measured TP loads by Osmond et. al. (2017) were lower in northwest Arkansas and the lower range of central Georgia.

The TP yield from subareas near the main stream was higher than that from other subareas (Fig. 6C). This elevated TP yield may be linked to the higher sediment load observed in these areas (Fig. 6B, C). Conversely, the lower TP yield from subareas 30 and 40, which are primarily urban (Fig. 2D), may be attributed to their lower sediment yield, as TP yield is positively correlated with sediment yield (Sharpley, 1985; Thapa et al., 2024; Thapa et al., 2025a; Wall et al., 1996). Another possible reason for the lower TP yield in subareas with predominantly residential land use is that limited application of phosphorus-based fertilizer, which are commonly used in agricultural areas and contribute to TP in streamflow.

The statistical indicators showed that measured and APEX-simulated daily TN yields were congruent. The R^2^, RMSE, NSE, PBIAS, and RSR values were 0.91, 0.033, 0.59, −21.33%, and 0.64, respectively, during the calibration process, and 0.83, 0.018, 0.56, 26.13%, and 0.67, respectively, during the validation process (Fig. 4D). The measured and simulated average annual TN yields were 5.40 kg/ha and 5.69 kg/ha, respectively. There was no significant difference between the measured and simulated average annual TN yields from the LRDW (*p* = 0.69; paired t-test; Fig. 5D). The TN load measured in this study was within the 4.1 to 11.9 kg/ha of TN yield reported by Daniels et al. (2019) from the cotton-producing farm in the lower Mississippi River basin in southeastern Arkansas. TN yield reported by Sharpley et al. (2020) in a field study in Arkansas was lower than that in this study, most likely due to the incorporation of a 30 m stream buffer at the downstream in the Sharpley et al. (2020).

TN yields were higher in subareas located near the main stream and in those predominantly covered by urban land use (Fig. 6D). Figures 6A and 6D also suggest a positive correlation between streamflow and TN yield (Daniels et al., 2019; Thapa et al., 2024; Thapa et al., 2025a). The elevated TN yields in these subareas may be attributed to the limited residence time for plants to uptake applied nitrogen fertilizers during rainfall or irrigation events (Zotarelli et al., 2008). In particular, the short flow paths from field (subarea) near the stream reduce the opportunity for plant filtration and absorption (Dillaha et al., 1988). Given the high solubility of nitrogen-based fertilizers, they are readily mobilized and transported with surface runoff (Aryal and Reba, 2017). Similarly, nitrogen originates from atmospheric deposition, anthropogenic activities (e.g., fertilizer application in gardens and lawns, pet waste, etc.), and organic materials (e.g., leaf litter, grass clippings, etc.). These sources add nitrogen to the urban runoff while also allowing less opportunity for nitrogen uptake due to high impervious surfaces, high hydrological connectivity, and reduced time of concentration in the nearby streams (Bell et al, 2019; Jani et al., 2020).

The scatter plots in Fig. 4 for daily pollutant yields indicate higher simulated streamflow, sediment, TP, and TN yields than the measured values. This is indicated by the regression lines in the scatter plots lying above the 1:1 (45°) line. Similarly, except for simulated streamflow, the box plots in Fig. 5 for average annual yields also showed that the simulated sediment, TP, and TN yields were higher than the measured pollutant yields. The negative PBIAS values for sediment and TP in the scatter plot also indicate that the simulated sediment and TP yields are overestimated relative to the measured values, and these differences are statistically significant (*p* < 0.05). In contrast, there were positive PBIAS values in the streamflow scatter plot, indicating that the simulated streamflow underestimated the measured streamflow, which is also verified by the boxplot. The significant overestimation of sediment and TP yield and non-significant underestimation of streamflow may be attributable to management and measurement data assumptions, as several categories of input data required to run the APEX model were assumed due to limitations in data availability.

### Uncertainty and reliability analysis results

A comprehensive analysis of the ArcAPEX model predictions was conducted using uncertainty and reliability assessments (Table 5). A negative e_avg_ indicates that the ArcAPEX model underestimated the observed values, whereas a positive e_avg_ indicates overestimation. Among all the predicted variables, streamflow showed the highest S*d*_*e*_, as well as the widest upper and lower confidence intervals (U95) due to the highest data magnitude as compared to the sediment, TP, and TN yield values. The results also showed that streamflow predicted by the ArcAPEX model was the most reliable as compared to sediment and TP yield predictions. Similar findings were reported by Kamruzzaman et al. (2020), Ramirez-Avila et al. (2017), and Senaviratne et al. (2018a). The lower reliability in sediment and nutrient yield simulations by the APEX model may be attributed to the use of user-defined runoff fractions of irrigation water and runoff depth, along with empirical relationships for erosion prediction, which can lead to overestimation of sediment and nutrient loads (Ramirez-Avila et al., 2017). Another possible factor is measurement error in streamflow, sediment, and nutrient yield data, as these yields were calculated on the streamflow, allowing potential error propagation during subsequent analyses. In addition, the use of dry-year data (2016 and 2017) for calibration and wet year data (2022) for validation may have affected sediment and nutrient yield predictions. Finally, the model’s limited ability to capture extreme events using the existing empirical equations may have contributed to its lower predictive accuracy, which warrants further investigation.

**Table 5:** Statistical performance of ArcAPEX model for streamflow andsediment, total phosphorus (TP), and total nitrogen (TN) yields based on average estimation errors (e_avg_), standard deviation of prediction error (Sd_e_), prediction confidence band (CL^+^- CL^-^), 95% uncertainty interval (U95), and reliability

Metrics	Streamflow	Sediment	TP	TN
e_avg_	0.1096	−0.0016	−0.0020	−0.0008
Sd_e_	1.8669	0.0210	0.0206	0.0284
CL_e_^+^	3.7688	0.0395	0.0383	0.0548
CL_e_^-^	−3.5496	−0.0427	−0.0423	−0.0564
Band	7.3183	0.0822	0.0806	0.1111
U95	0.1194	0.0015	0.0008	0.0019
Reliability	18.14%	6.33%	7.84%	7.80%

*CL*^*-*^ lower confidence limit, *CL*^*+*^ upper confidence limit

Although lower uncertainty (U95) and higher reliability values are generally preferred for accurate model performance which typically reduces random errors in the measurement process (Saberi-Movahed et al., 2020), the ArcAPEX model in this study achieved uncertainty values for streamflow and sediment, TP, and TN yields were 0.119 mm, 0.0015 ton/ha, 0.0008 kg/ha, and 0.0019 kg/ha, respectively. The calculated unreliability values were very small compared to the measured and simulated values (Fig. 4 and Table 5), which strengthens the accuracy and predictive capability of the ArcAPEX model. The range of reliability values of this study for streamflow, sediment, TP, and TN yields predictions fall between 5.17% reported by Ekambara and Joshi (2003), 25.86% reported by Hart et al. (2013), and 12.07–25.86% reported by Saberi-Movahed et al. (2020). Notably, the uncertainty and reliability in streamflow prediction was comparable to the values reported by Saberi-Movahed et al. (2020), suggesting the robustness of the ArcAPEX model in simulating hydrological processes.

### Effects of filter strips and cover crops implementation scenarios on pollution yield reduction

The impact of cover crops, filter strips, and their combined application on streamflow, sediment, TP, and TN yield reductions were evaluated under different application scenarios. For these purposes, cover crops were implemented by adding rye grass as a cover crop in the management section of the calibrated APEX baseline model. Similarly, filter strips were implemented by modifying subarea parameters in the subarea section and land use numbers in the management section for kentucky 31 tall fescue grass.

**Fig. 7:**
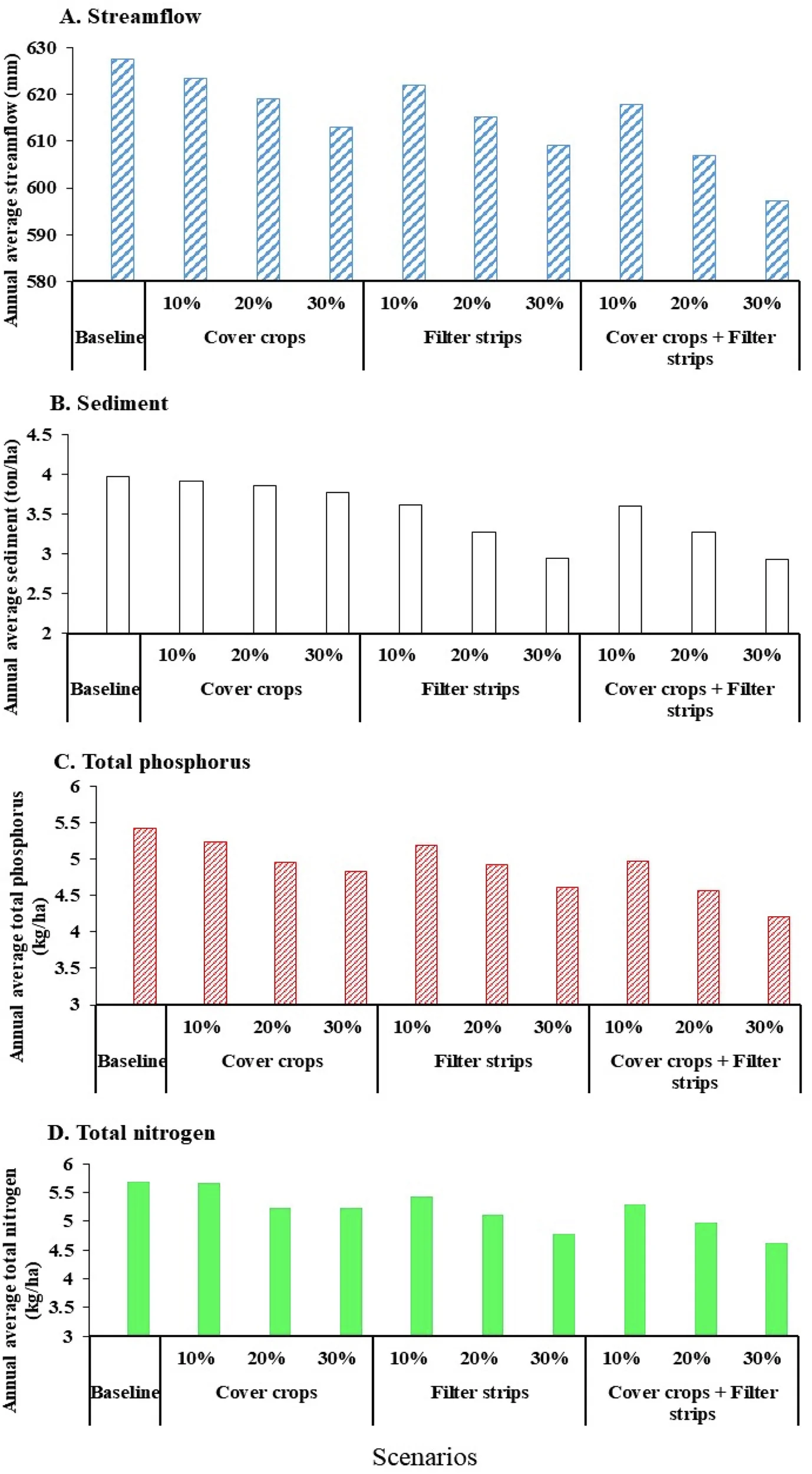
Comparison of annual average streamflow (**A**), and annual average sediment (**B**), total phosphorus (**C**), and total nitrogen (**D**) yields from the Little River Ditches watershed under the baseline (existing negligible implementation of cover crop and filter strips) and under conservation practice scenarios with 10%, 20%, and 30% of the watershed area implemented with cover crops and filter strips individually and in combination after running ArcAPEX model using data from 2015 to 2022

The simulated APEX model baseline results showed that the annual average streamflow was 627 mm (Fig. 7A). Implementing cover crops led to streamflow reductions ranging from 0.6% (623 mm) at 10% coverage to 2.3% (613 mm) at 30% coverage within the watershed. Filter strips demonstrated a slightly larger reduction, ranging from 1% (622 mm) at the subarea representing 10% coverage in the watershed to 2.94% (618 mm) at the subarea representing 30% coverage. The combination of cover crops and filter strips achieved the greatest streamflow reduction, up to 4.82% (597 mm) at 30% subarea coverage in the watershed. The streamflow reduction percentage was higher when considering specific subareas rather than the entire watershed. Results indicated that evapotranspiration from fields with cover crops was much greater and the percolation was less compared to that without cover crops. Consequently, streamflow was lower when cover crops were implemented due to increased evapotranspiration. For the filter strips, changes in land use numbers, erosion control practice factors, and channel properties altered surface hydrology, resulting in less streamflow in some subareas of the watershed.

The APEX model baseline study revealed that the annual average sediment yield from the watershed was 3.96 tons/ha (Fig. 7B). Sediment yield was notably reduced with the use of cover crops, filter strips, and combination of both. The introduction of cover crops alone resulted in sediment yield reductions ranging from 1.42% (3.91 tons/ha) at 10% cover crop areas in the watershed to 4.85% (3.77 tons/ha) at 30% cover crop areas in the watershed. Filter strips exhibited a more substantial sediment yield reduction. Sediment yield reductions ranged from 9.01% (3.91 tons/ha) with filter strips implemented in the subareas that represented 10% of the watershed to 25.71% (2.94 tons/ha) with filter strips implemented in the subareas that represented 30% of the watershed. The combination of both cover crops and filter strips further enhanced sediment yield reduction. When the cover crop and filter strips area accounted for 10%, 20%, and 30% of the area in the watershed, the sediment yield was reduced by 9.1% (3.6 tons/ha), 17.67% (3.26 tons/ha), and 26% (2.93 tons/ha), respectively. The reduction of sediment yield by cover crops might be due to the reduction in runoff due to changes in hydrology. The cover crops and filter strips enhanced transpiration, infiltration, and soil moisture retention, slowed the peak flow rate, and trapped sediment by standing plants from the runoff, ultimately reducing the sediment yield to the stream from the watershed (Blanco-Canqui et al., 2011; Reeves, 2018; Thapa et al., 2024).

The watershed’s annual average TP yield was estimated to be 5.42 kg/ha by the baseline APEX model (Fig. 7C). TP was reduced by 3.34% (5.24 kg/ha) to 10.8% (4.83 kg/ha) when cover crops were implemented in 10% and 30% areas of the watershed. Filter strips exhibited slightly better TP reductions, ranging from 4.38% (5.18 kg/ha) to 15% (4.6 kg/ha) when filter strips were implemented in 10% and 30% of the watershed area. The combination of cover crops and filter strip treatment for 30% of the watershed area resulted in the highest percentage reduction in TP yield, achieving a 22.4% reduction (4.2 kg/ha). The TP yield reduction by cover crop and filter strips was likely due to the uptake of phosphorus as a nutrient by plants and nutrient cycling in the field, as well as the reduction of sediment-bound phosphorus due to the reduction of sediment yield from the field (Thapa et al., 2024).

In the baseline study, the APEX model simulated average annual TN yield at 5.69 kg/ha in the baseline study (Fig. 7D). The cover crop reduced the TN yield by 2.45% (5.22 kg/ha) when the cover crop was implemented in 30% of the watershed area. Filter strips showed greater effectiveness, with a reduction ranging from 4.47% (5.43 kg/ha) to 16% (4.78 kg/ha) when filter strips were planted in the subarea, representing 10% and 20% of the watershed area. The combined treatment of the cover crop and filter strips provided the highest nitrogen reduction. The percentage TN reduction was 6.96% (5.29 kg/ha), 12.71% (4.96 kg/ha), and 18.95% (4.61 kg/ha) when cover crops and filter strips were planted in 10%, 20%, and 30% of the watershed. The reduction in TN by cover crops and filter strips was attributed to plant uptake, which cycled within the field. These practices trapped sediment-bound nitrogen, thereby reducing TN load in the streams (Thapa et al., 2024).

The percentage reduction of different pollutant species increased with greater coverage of cover crops, filter strips, and their combination. This study found the optimum reduction at 30% coverage, which achieved the highest pollutant yield reduction. The results also indicated that while cover crops contributed to reducing streamflow and sediment and nutrient yields, filter strips alone provided more benefits, particularly in reducing sediment and phosphorus yields. The differences may be due to greater soil surface disturbance from tillage activities when implementing cover crops. The combined application of cover crops and filter strip practices achieved the highest streamflow, sediment, TP, and TN yield reduction. Therefore, combined applications would be the most effective strategy for mitigating streamflow, sediment, and nutrient yield to agricultural streams while fostering soil conservation.

Sediment yield reduction was the greatest, followed by reductions in TP, TN, and streamflow in treatments involving cover crops, filter strips, and their combination in APEX simulation. The highest percentage reduction was observed for sediment yield because cover crops and filter strips effectively trapped solid sediment particles. This mechanism might have been reflected in erosion control practice factors (PEC), land use number (LUN), and Manning’s number, which were changed. During conservation practice simulations, streamflow reduction was lower in percentage than sediment yield reduction. This might have led to lower percentages of TP and TN reduction since these nutrients are highly soluble in the streamflow and may be less bound with sediment particles, even though these are also directly proportional to the sediment load (Thapa et al., 2024).

### Limitations of this study

When watershed delineation was performed using ArcAPEX within the ArcGIS interface with a 10 m DEM, some portions of the delineated reach channels or drainage ditches, or stream network, did not align with the existing drainage ditches in the watershed. In such cases, manual delineation of reach channels was performed using the masking and burn-in functions in ArcAPEX to better align with the existing drainage network. However, small channels were not corrected because small field channels were created by the topography of the field rather than by constructed ditches. The discrepancy between the delineated reach channels and the existing drainage network during the ArcAPEX delineation process may be attributed to the coarse resolution of the DEM or the relatively flat nature of the watershed, which limits the detection of narrow drainage ditches. This highlights the challenges of delineating flat agricultural watersheds and the need for a very fine-resolution DEM.

While the daily streamflow was calculated from the 15-minute interval at field-collected data, daily sediment and nutrient yield were estimated from the weekly sediment and nutrient concentrations in water samples, which may not reflect the actual sediment, TP, and TN yields. The streamflow, sediment, TP, and TN yield from each subarea were not measured in the field and could not be verified with the simulated streamflow, sediment, TP, and TN yield at the subarea level. However, we verified the measured and simulated pollutant yield at the watershed level.

## Conclusions

The study, conducted from 2015 to 2022 in a 53.78 km² (13,289 ac) predominantly agricultural watershed within the Little River Ditches Watershed (LRDW) in Arkansas, applied the GIS-based Agricultural Policy Environmental eXtender (ArcAPEX) model to simulate streamflow, sediment, total phosphorus (TP), and total nitrogen (TN) yields. The results demonstrated that the ArcAPEX model is an effective tool to simulate streamflow, sediment, TP, and TN yields from agricultural watersheds, as the performance statistics observed for simulated and measured streamflow, sediment, TP, and TN yields were acceptable (R^2^ > 0.8, NSE > 0.5, |PBIAS | < 30%, RSR < 0.7) with less uncertainty and higher reliability. The average annual measured and simulated streamflow and sediment, TP, and TN yields from the Little River Ditches Watershed were 668 mm and 628 mm, 3.39 tons/ha and 3.97 tons/ha, 4.69 kg/ha and 5.41 kg/ha, and 5.4 kg/ha and 5.69 kg/ha, respectively, with no significant differences between measured and simulated streamflow and TN yields (*p* > 0.05). The model also indicated that subareas near the main stream produced higher streamflow, sediment, TP, and TN yields, while urban subareas had lower sediment and TP yields but higher streamflow and TN yields.

Scenario-based simulations revealed that implementing cover crops and filter strip treatments individually or in combination across 10% to 30% area of the watershed effectively reduced streamflow, sediment, TP, and TN yields. The findings confirmed that the combination of cover crops and filter strips in the watershed was more effective than either cover crops or filter strips implementation, highlighting the value of integrated conservation strategies. Consequently, efforts by agricultural policymakers and farm managers to increase the adoption of multiple conservation practices at the field, farm, and watershed levels are needed to abate agricultural water pollution. Sharing the calibrated parameters and detailed calibration approach enhances model reproducibility and transferability to other agricultural watersheds, supporting comparative modeling and management efforts. Future research should focus on evaluating multiple agricultural conservation practices and their effects on instream and downstream water quality simultaneously under varying climate and management conditions, coupling surface water-groundwater models to better capture hydrological interactions, and incorporating socio-economic assessments to evaluate the feasibility and cost-effectiveness of conservation strategies. Such studies will strengthen integrated watershed management and inform evidence based on agricultural policy development.

## Data Availability

Data will be available in Ag Data Commons https://agdatacommons.nal.usda.gov/.
